# Dynamic Responses of the Caudal Neurosecretory System (CNSS) Under Thermal Stress in Olive Flounder (*Paralichthys olivaceus*)

**DOI:** 10.3389/fphys.2019.01560

**Published:** 2020-01-09

**Authors:** Mingzhe Yuan, Xiaoxue Li, Tianyi Long, Yan Chen, Weiqun Lu

**Affiliations:** ^1^National Demonstration Center for Experimental Fisheries Science Education, Shanghai Ocean University, Shanghai, China; ^2^The Key Laboratory of Exploration and Utilization of Aquatic Genetic Resources, Ministry of Education, Shanghai, China; ^3^International Research Center for Marine Biosciences at Shanghai Ocean University, Ministry of Science and Technology, Shanghai, China

**Keywords:** caudal neurosecretory system, corticotrophin-releasing hormone, urotensin I, urotensin II, thermal stress, fish

## Abstract

Temperature is a critical environmental factor that affect most biological and physiological processes in fish. The caudal neurosecretory system (CNSS) is unique to fish and is proved to maintain homeostasis during seasonal alterations. However, the dynamic expression and secretion pattern of its major hormones, corticotrophin-releasing hormone (CRH), urotensin I (UI), and urotensin II (UII), and their response to thermal stress has not been studied. CRH, UII and cortisol in plasma, gene expression levels of CRH, UI, and UII in the CNSS of olive flounder (*Paralichthys olivaceus*) were therefore characterized. UI- and UII-positive Dahlgren cells, as well as cell proliferation in the CNSS, were also quantified. The results showed that plasma cortisol and CRH were increased in both low temperature (LT) and high temperature (HT) groups. However, there was no difference in plasma UI and UII during thermal stress. In CNSS, CRH, UI, and UII mRNA levels were all significantly elevated in response to acute hypothermal stress and recovered back to the control (normal) level after 8 days of adaptation. During hyperthermal challenge, gene expression of CRH and UI only significantly increased after 8-days of transfer but no change in UII was observed. We also demonstrated an increasing percent of UI-positive Dahlgren cells in the CNSS of 8-days hyperthermal stressed fish. However, no BrdU-labeled Dahlgren cells were found among the three treatment groups. Collectively, our results demonstrate that the CNSS is subjected to dynamic responses under thermal stress and expands upon the role of the CNSS in thermoregulation. The dynamic responses of hormone levels and the gene expression of CRH, UI and UII in CNSS are all involved in the process of hyper- or hypo-thermal stress and adaptation.

## Introduction

Temperature is one of the critical abiotic factors that would affect many aspects of animals. Active thermoregulation inherent in homeothermic animals is important to preserve molecular and cellular functions critical for life (Nakamura and Morrison, 2010). In addition, almost all fish, with the exception of a few large pelagic fish (e.g., tuna, mako sharks), lack the ability to retain endogenous heat because the gills and body surface consistently and rapidly exchanges heat with the surrounding environment (Stevens and Sutterlin, 1976). Therefore, fish cannot maintain a constant body temperature that is different from the external environment meaning that temperature is a critical abiotic factor that affects most biological and physiological processes, including growth, reproduction, digestive ability, and immune function (Hazel and Prosser, 1974; Marley et al., 2008). When fish encounter an acute or long-term change inoptimal temperature, homeostatic responses and energetic reorganization are needed for survival in allostatic conditions (Juriaan et al., 2003; McEwen and Wingfield, 2003). In fish, neuroendocrine signaling affects and becomes regulated by the onset of immune responses, due to the peculiar organization of the head kidney, a hematopoietic tissue made from a mixture of endocrine, hematopoietic and immune cell populations. Besides that, responses could also be mediated by the activation of two hormonal axes in fish, the Sympatho-Chromaffin (SC) axis and the Hypothalamic-Pituitary-Interrenal (HPI) axis (Carles Balasch and Tort, 2019).

Cortisol, a common global marker of the stress, acts as a regulatory component of neuro-immuno-endocrine circuitry by binding to glucocorticoid (GR) or mineralocorticoid (MR) receptors, eliciting stress-induced immunosuppression and allostatic imbalances (Benitez-Dorta et al., 2017). Plasma cortisol is traditionally regulated by a signal pathway of corticotrophin-releasing hormone (CRH), adenocorticotropin hormone (ACTH), within the HPI axis (Nesan and Vijayan, 2016). However, there is an additional set of neuroendocrine cells in the caudal neurosecretory system (CNSS), which can release CRH into the circulation of fish. The CNSS is located in the terminal segments of the spinal cord, including magnocellular peptide-synthesizing neuroendocrine neurons (Dahlgren cells) and a neurohemal organ (the urophysis), in which synthesized peptides can be stored and released into circulation from the capillaries of the caudal vein (Winter et al., 2000; Lu et al., 2004, 2006).

The CNSS is the major circulating source of CRH, Urotensin I (UI), and Urotensin II (UII) in fish (Lu et al., 2004, 2006). UI is a 41-amino acid peptide (Lederis et al., 1982) belonging to the superfamily of CRH, which also includes the mammalian UI ortholog, urocortin (Lovejoy and Balment, 1999; Lu et al., 2004). UII is a cyclic peptide formed by a disulfide bond at the C terminus, in which the “-Cys-Phe-Trp-Lys-Tyr-Cys-” sequence is fully conserved across vertebrate species (Lu et al., 2006). CRH and UI are known to regulate and integrate the neuroendocrine, autonomic, immune, and behavioral response of fish to stressors by affecting stress-related cortisol production (Kelsall and Balment, 1998; Craig et al., 2005). Additionally, UII is found to maintain body fluid homeostasis by regulating water and ion transport (Lu et al., 2006). Therefore, the CNSS is proposed to play a role in many aspects of adaptive physiology, including osmoregulation, reproduction, nutrition and stress-related responses (Winter et al., 2000; Lu et al., 2004, 2006). Previous studies have found that the CNSS might be functionally reprogramed to cope with changes in physiological challenge during seasonal alternation (Lu et al., 2007; Chen and Mu, 2008). As temperature is arguably the most critical seasonal factor, the role that the CNSS plays in thermal stress and adaptation should be resolved (Mccrohan and Bernier, 2007).

The olive flounder (*Paralichthys olivaceus*) is a marine demersal species found along the coasts of Japan, Korea, and China (Sabate et al., 2008; Tomiyama et al., 2008). This flounder migrates from deep (cold) seas to shallow (warm) areas for reproduction, so thermoregulation is an important way to keep their internal environment steady. In order to better understand the physiological roles of CNSS in thermoregulation, we investigated the dynamic changes in CRH, UI and UII peptides, gene expression levels, as well as the cellular level in CNSS during thermal stress. We hypothesized that the adaptability of the olive flounder to thermal stress is associated with the dynamic response of CRH, UI, and UII in the CNSS.

## Materials and Methods

### Fish and Ethics Approval

The gynogenetic olive flounder is a very popular fish for aquaculture in China. Sexual dimorphisms are potentially seen when fish are subject to stress so gynogenetic fish were used in this study. Gynogenetic olive flounder were produced as previously described (Liu et al., 2012) and reared in recirculating aquaculture systems at the Central Experimental Station of Chinese Academy of Fisheries Sciences (Beidaihe, China). The experiment was conducted in September, 2016. A total of 108 gynogenetic olive flounders (body weights: 500 ± 50 g) were randomly allocated to 24 tanks with flow-through, filtered seawater (30‰) systems at 18 ± 1°C for more than 2 weeks. Black plastic light-proof curtains surrounded each set of tanks and artificial illumination was provided with white fluorescent lamps. Mean light intensity was approximately 40 lux measured centrally at the bottom of each seawater tank. Fish were not fed during the experiment in order to reduce the influence of feeding. The experimental protocol was approved by the Institutional Animal Care and Use Committee (IACUC) of Shanghai Ocean University (SHOU), Shanghai, China, and abides by the Guidelines on Ethical Treatment of Experimental Animals established by the Ministry of Science and Technology, China.

### Thermal Stress Experiments

Fish were divided into six treatment groups: (1) low temperature group (LT, 12°C; *n* = 24), (2) normal temperature group (NT, 18°C; *n* = 24), (3) high temperature group (HT, 24°C; *n* = 24), (4) low temperature group injected with 5′-bromo-2′-deoxyuridine, BrdU (LT + BrdU, 12°C; *n* = 12), (5) normal temperature group injected with BrdU (NT + BrdU, 18°C; *n* = 12), (6) high temperature group injected with BrdU (HT + BrdU, 24°C; *n* = 12). In the BrdU treatment groups, fish were injected intraperitoneally with a single dose of saline-BrdU (Sigma, Germany; 0.2 mg/g body weight) solution within 30 s before the fish were transferred into the corresponding tank. All fish were acclimated in each experimental condition for 8 days and sampled during daytime from 10 am to 3 pm at 2 h, 1, 2, and 8 days after transfer. Fish were removed from each time point tank and, without using anesthetic, blood samples (3–5 ml) were collected within 90 s into ammonium-heparinized syringes by caudal venepuncture. Blood was aliquoted into ammonium-heparinized tubes and plasma separated by centrifugation for 5 min at 13,000 × *g* and stored at −80°C until the measurement of plasma hormones by ELISA. Fish were then humanely killed using spinal cord severance and brain excision. The CNSS of the fish from the LT, NT and HT groups were removed and instantly frozen in liquid nitrogen for subsequent analysis of gene expression. The CNSS from BrdU treatment groups were removed and stored in 4% paraformaldehyde (PFA) at 4°C. All samples were taken during daylight hours.

### Plasma Measurements

Plasma levels of CRH, cortisol (COR), and UII were quantified by ELISA commercially (Qiyi Biotechnology Co., Ltd., Shanghai, China). According to the manufacturer’s instructions, the circulating level range of COR was detected between 100 and 1800 ng/L, CRH was detected between 15 and 900 ng/L and UII was detected between 3 and 120 ng/L. In detail, −80°C stored plasma supernatant fractions were naturally warmed in an ice box. Samples were then diluted to the appropriate concentration. Commercially available ELISA kits (Qiyi Biotechnology Co., Ltd., Shanghai, China) were subsequently used to measure serum COR, CRH, and UII levels in duplicate as per manufacturer instructions (Mechesso et al., 2019).

### Relative Quantitative RT-PCR

The CNSS mRNA expression levels were analyzed by quantitative real-time PCR on ABI 7500 Real-Time PCR System (Applied Biosystems, Singapore). Relative quantification of the target gene transcripts was analyzed using β-actin gene expression as the reference gene (Yuan et al., 2017). Sequences of CRH, UI, UII were obtained from GeneBank. The primers were designed using Primer Premier 5 software (PREMIER Biosoft International, Palo Alto, CA, United States), and synthesized commercially (Sangon Biotech, Shanghai, China) (Table 1). The optimization and validation of primers and probes were performed using standard ABI protocols.

**TABLE 1:** Gene specific primers for β-actin, CRH, UI, and UII of olive flounder *P. olivaceus*.

**Gene**	**GeneBank**	**Primers (5′–3′)**
β-actin	HQ386788.1	F: GGAAATCGTGCGTGACATTAAG
		R: CCTCTGGACAACGGAACCTCT
CRH	XM_020087578.1	F: AAAGGAGGTGAAGGAGGA
		R: AAGAAGGCAACAAGCAGA
UI	XM_020105024.1	F: GACCTGCTGAGCGACAA
		R: TCATCCTCGGCTATCTGG
UII	XM_020096040.1	F: ATCTGCTGAGATGCCCTATC
		R: CTGTTGTTCTCCACCGTCTC

Total RNA was extracted from the tissues of each individual by RNAiso Plus (TaKaRa, Japan). One microgram of total RNA was treated by PrimeScript^TM^ RT reagent kit with gDNA Eraser. Briefly, quantitative real-time PCR assays were run using FastStart Universal SYBR Green Master kit (Roche, United States), in 20 μl reaction volume, under a standard amplification procedure (2 min at 50°C, 10 min at 95°C and then 40 cycles of the following process: 15 s at 95°C and 30 s at 60°C).

### UI, UII, and BrdU Immunofluorescence Chemistry

Paraformaldehyde-fixed CNSS were dehydrated in ethanol, cleared in xylene and embedded in paraplast. Every section (5-μM-thick) was cut on a microtome and mounted on glass slides with a positive charge. Briefly, tissue sections were dewaxed in xylene and rehydrated in gradient alcohol. Endogenous peroxidase activity was blocked with 3% H_2_O_2_ in methanol before slides were placed in 0.01M citrate buffer and heated in a water bath for 20 min at 95°C. After cooling, sections were rinsed in PBS. For UI and UII immunofluorescence chemistry, sections were treated with fetal bovine serum (FBS) blocking solution (1% blocking, dissolved in MABT, and 5% FBS in PBST, PBS with 0.1% Triton X-100) for 1 h at room temperature (RT) to reduce non-specific staining and incubated with 1:500/1:1000 rabbit anti-UI/UII antibodies [produced by Lu et al. (2004, 2006)] diluted with PBS in a moist chamber at 4°C overnight. The moist chamber was transferred into an air oven at 37°C for 45 min, then washed six times at RT in PBST for 15 min each time and incubated with 1:100 DAPI and 1:500 goat anti-rabbit IgG (H + L) highly cross-adsorbed secondary antibody, Alexa Fluor Plus 488 (Thermo Fisher Scientific, United States), diluted with PBS for 1 h in dark. The slides were washed six times in 5% FBS (in PBST) for 90 min. For BrdU immunofluorescence, sections were incubated in 2 N HCl for 30 min at RT, followed by thorough washing in PBS and blocked with 5% FBS in PBST for 1 h at RT. Sections were incubated with 1:100 mouse anti-BrdU antibody (Sigma-Aldrich, Germany) diluted with PBS in a moist chamber at 4°C overnight. After thorough buffer rinses, the sections were incubated with 1:100 DAPI (Sangon Biotech, Shanghai, China) and 1:500 goat anti-mouse IgG (H + L) highly cross-absorbed secondary antibody, Alexa Fluor Plus 568 (Thermo Fisher Scientific, United States), diluted with PBS for 1 h in dark. Then the sections were washed three times in PBST. Control experiments were carried out by omission of the primary antibody and preabsorption of the antibody with an excess of antigenic peptide.

### Quantification of Labeled Cells

Single-labeling UI/UII and BrdU experiments of short and long term thermal stress were used for quantification of protein secretion and cell proliferation. Cell counts of UI/UII and BrdU labeling were obtained within each CNSS in every 20 sections from all sections sampled (5 μm-spaced serial sections parallel to the entire central canal axis of the CNSS) at 400× magnification with a fluorescence microscope (Nikon ECLIPSE 55i, Nikon Corporation, Japan). The UI-/UII- and BrdU-positive cells, as well as all Dahlgren cells, were counted. The numbers of UI-/UII-positive Dahlgren cells were expressed as the proportion of total Dahlgren cells, while the numbers of BrdU-positive cells were counted as the fraction of BrdU-positive cells per unit area of the CNSS. Size and luminosity of the figures were modified with NIS-Elements Version 4.0 (Nikon, Japan). The graphs and drawings were prepared using Adobe PhotoshopCS 6 (Adobe System, United States).

### Statistics

The 2^–ΔΔ*Ct*^ method was used to analyze the real-time PCR data (Livak and Schmittgen, 2001), and amplified transcripts were expressed as the fold change relative to the mean value of the standard sample. Data were tested for normality by the Shapiro–Wilk’s test and homogeneity of variance by Levene’s test, and expressed as means ± SEM. Significant analyses were conducted by two-way ANOVA with treatment and time as independent variables, followed by the Tukey’s multiple comparison test when changes in data were assessed for each treatment or time. Significant effects of temperature treatment in BrdU positive cells were conducted by one-way ANOVA with Dunnett’s test. Results were considered significantly different when *P* < 0.05. All analyses were conducted using a computer program, GraphPad Prism 5.0 (San Diego, CA, United States).

## Results

### Plasma Hormones

**FIGURE 1:**
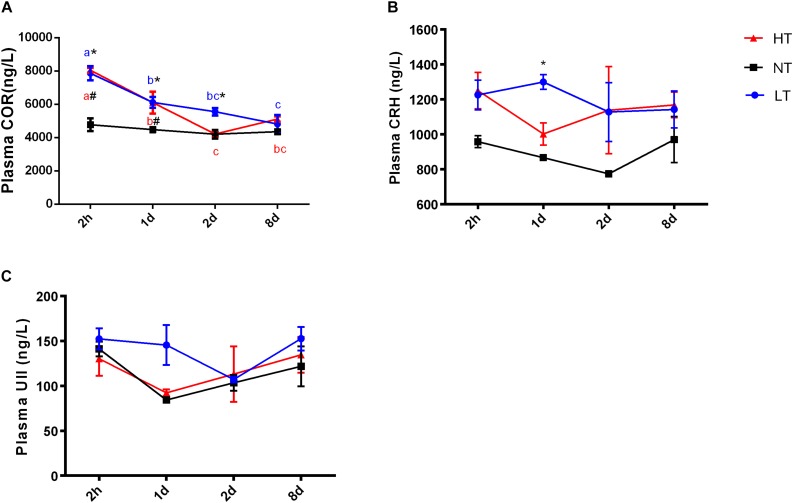
Dynamic changes in plasma cortisol **(A)**, CRH **(B)**, and UII **(C)** after transfer of olive flounder from NT to LT (•) or HT (▲). Mean ± SEM (*n* = 6). Control fish were maintained in NT and sampled across the same time course (■). Significant analyses were conducted by two-way ANOVA with treatment and time as independent variables, followed by the Tukey’s test. Results were considered significantly different when *P* < 0.05. Symbol “^∗^” or “#” was used to represent the significant difference between NT and LT or HT within a time point. Different small letters were used to represent a significant difference across time points within a treatment group.

Significant interactions between temperature and time were only detected for cortisol concentrations in plasma [two-way ANOVA, *F*(6,30) = 6.677, *P* = 0.0027], indicating that stress hormone responses were different among the temperature treatments at different time points. After 2 h thermal stress, plasma cortisol was significantly increased to the highest level in both LT (*P* = 0.0075) and HT (*P* = 0.0036) groups. Subsequently, cortisol levels declined significantly with time in both HT (Tukey’s test, 2 h vs. 1 day: *P* = 0.0036; 2 h vs. 2 days: *P* < 0.0001; 2 h vs. 8 days: *P* < 0.0001; 1 day vs. 2 days: *P* = 0.0042) and LT (Tukey’s test, 2 h vs. 1 day: *P* = 0.0075; 2 h vs. 2 days: *P* = 0.0009; 2 h vs. 8 days: *P* < 0.0001; 1 day vs. 8 days: *P* = 0.0472) treatments (Figure 1A). Plasma CRH level increased but not significantly more than the NT group in both LT and HT groups during thermal stress treatments. A statistically significant increase of CRH level was only found in LT group at 1 day (*P* = 0.0058, Figure 1B). There was no remarkable difference with time or temperature treatments for plasma UII during the experiment (Figure 1C).

### CRH, UI, and UII Gene Expression in CNSS

Significant interactions between temperature and time were observed for CRH [two-way ANOVA, *F*(6,30) = 3.51, *P* = 0.0123], UI [two-way ANOVA, *F*(6,30) = 6.045, *P* = 0.0006] and UII [two-way ANOVA, *F*(6,30) = 13.08, *P* < 0.0001] gene expression in CNSS, indicating that gene expression of CRH, UI and UII in CNSS was different among the temperature treatments at different time points. The expression of these three genes was therefore compared between the different thermal stress and control treatments at each sampling time, and changes in gene expression were assessed for each thermal stress.

**FIGURE 2:**
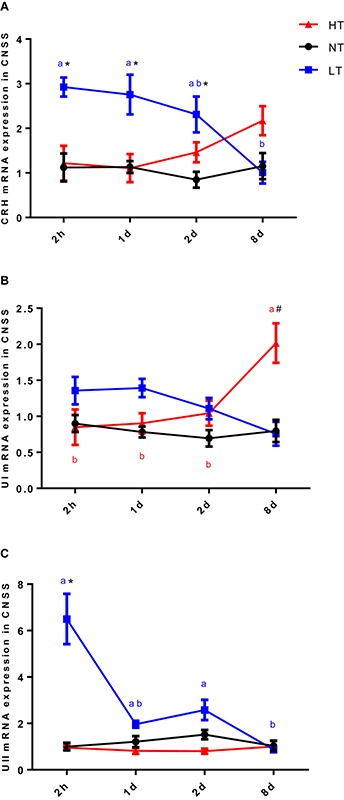
Dynamic changes in gene expression of CRH **(A)**, UI **(B)**, and UII **(C)** after transfer of Olive flounder from NT to LT (•) or HT (▲) in CNSS. Mean ± SEM (*n* = 6). Control fish were maintained in NT and sampled across the same time course (■). Significant analyses were conducted by two-way ANOVA with treatment and time as independent variables, followed by the Tukey’s test. Results were considered significantly different when *P* < 0.05. Symbol “^∗^” or “#” was used to represent the significant difference between NT and LT or HT within a time point. Different small letters were used to represent a significant difference across time points within a treatment group.

Two hours after hypothermia, the gene expression of CRH and UII in CNSS was significantly higher than the control group, but UI did not show a statistically significant increase (Tukey’s test, CRH: *P* = 0.0054, UII: *P* < 0.0001). Subsequently, gene expression of CRH (Tukey’s test, 8 days vs. 2 h: *P* = 0.0058; 8 days vs. 1 day: *P* = 0.0127) and UII (Tukey’s test, 2 h vs. 1 day: *P* < 0.0001; 2 h vs. 2 days: *P* < 0.0001 2 h vs. 8 days: *P* < 0.0001; 2 days vs. 8 days: *P* = 0.0272) declined significantly with time (Figures 2A–C). In contrast, the gene expression of UI showed a remarkable increase in CNSS 8 days after hyperthermia (Tukey’s test, *P* = 0.0001) with a significant increase with time (Tukey’s test, 8 days vs. 2 h: *P* = 0.0004, 8 days vs. 1 days: *P* = 0.0007 8 days vs. 2 days: *P* = 0.003; Figure 2B). There was no significant difference with time in CRH and UII mRNA expression during the experiment (Figures 2A,C).

### Count of UI and UII Positive Dahlgren Cells in CNSS

**FIGURE 3:**
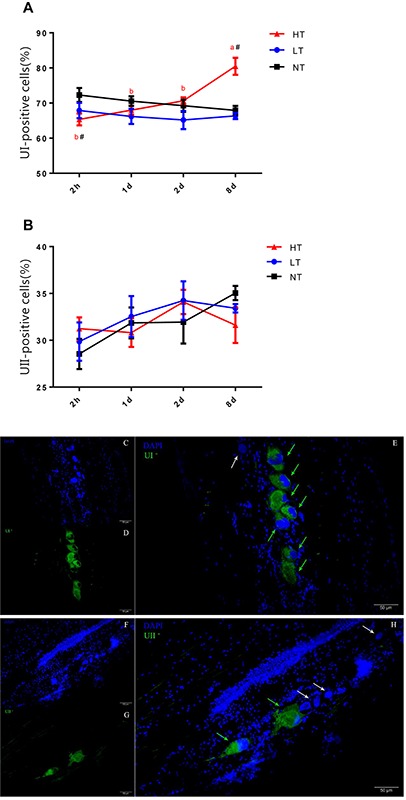
Dynamic changes in percent of UI- **(A)** and UII- **(B)** positive Dahlgren cells after transfer of olive flounder from NT to LT (•) or HT (▲) in CNSS. Mean ± SEM (*n* = 3). Control fish were maintained in NT and sampled across the same time course (■). Sagittal paraffin sections of CNSS after treatment with anti-UI **(D)** and UII **(G)**, revealed by FITC. Nucleus were stained by DAPI **(C,F)**. The superimposition of two labeled signals was conducted by computer **(E,H)**. Green and White arrows pointed out the labeled and unlabeled Dahlgren cells, respectively. Significant analyses were conducted by two-way ANOVA with treatment and time as independent variables, followed by the Tukey’s test. Results were considered significantly different when *P* < 0.05. Symbol “^∗^” or “#” was used to represent the significant difference between NT and LT or HT within a time point. Different small letters were used to represent a significant difference across time points within a treatment group.

Since UI and UII were produced by Dahlgren cells in CNSS, a count of UI and UII positive Dahlgren cells could represent the storage and secretion level of these two hormones in the CNSS. Dahlgren cells were distributed in the spinal cord dorsal to the 1st–6th preterminal vertebrae and appeared laterally and ventrolaterally to the central canal in olive flounder (Figures 3C–H). Significant interactions between temperature and time were measured for the percent of UI-positive Dahlgren cells [two-way ANOVA, *F*(6, 12) = 5.412, *P* = 0.0064]. The percent of UI-positive Dahlgren cells showed a remarkable increase in CNSS after 8 days of hyperthermia (Tukey’s test, *P* = 0.0017) with a significant increase over time (Tukey’s test, 8 days vs. 2 h: *P* = 0.0007; 8 days vs. 1 day: *P* = 0.0032; 8 days vs. 2 days: *P* = 0.0173; Figure 3A). For the LT group, the percent of UI-positive Dahlgren cells did not show any difference with time. Meanwhile, the percent of UII-positive Dahlgren cells increased. But, it had no significant effect of temperature or time (Figure 3B).

### Count of BrdU Positive Cells in CNSS

**FIGURE 4:**
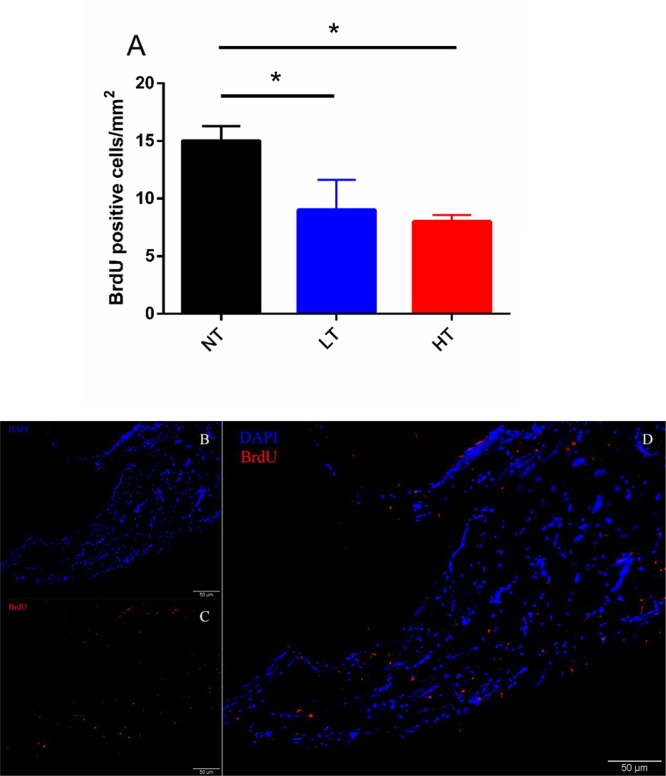
Changes in BrdU positive cells after 8-days transfer of Olive flounder from NT to LT or HT in CNSS **(A)**. Mean ± SEM (*n* = 3). Sagittal paraffin sections of CNSS after treatment with anti-BrdU **(B)**, revealed by TRITC. Nucleus were stained by DAPI **(C)**. The superimposition of two labeled signals was conducted by computer **(D)**. Control fish were maintained in NT and sampled across the same time course. Differences among groups were evaluated by one-way ANOVA with Dunnett’s test. Symbol “^∗^” was used to represent a significant difference between NT group (*P* < 0.05).

According to the changes of UI and UII positive Dahlgren cells in CNSS, BrdU positive cells in CNSS were counted after 8 days of thermal stress. However, no BrdU-positive Dahlgren cells were observed in either LT or HT treatment groups. BrdU-labeled cells were found near the central canal (Figures 4B–D). Compared to the NT group a significant decrease in BrdU-positive cells was found in both LT (Dunnett’s test, *P* = 0.0491) and HT (Dunnett’s test, *P* = 0.0244) groups (Figure 4A).

## Discussion

When fish encounter an acute thermal stress, as with many other stressors, stress-related physiological responses are immediately activated. For instance, the stimulation of the HPI axis elicits an increase in plasma cortisol. Therefore, previous studies had generally put more attention on stress-related regulation of CRH and UI in the brain (Pepels et al., 2004; Pinkel et al., 2007). However, there is an additional area in central nervous system (CNS) which is an important source of CRH, UI and UII in fish, the CNSS, but its function still remains unclear. In this study, we elaborated the dynamic changes of cortisol, CRH and UII in plasma, as well as their expression, changes in UI:UII Dahlgren cell ratio and the rate of cell proliferation in the CNSS during thermal stress. Overall, our results confirmed that there was a dynamic response of hormone levels and gene expression of CRH, UI and UII in CNSS associated with hyper- and hypo-thermal stress and thermal adaptation.

Using the ELISA kits to analyze the plasma cortisol in olive flounder, previous study showed that the plasma cortisol level in control group was around 5 ng/mL which was similar with our results (around 4 ng/mL) (Mechesso et al., 2019). Comparing with the results of RIA, the control level of cortisol was around 8 ng/mL (Zou et al., 2018). It had also been found that acute stress elevated plasma cortisol levels within minutes to hours (Pavlidis et al., 2015) but plasma cortisol declined back to pre-stress levels after long-term acclimation (Hosoya et al., 2007). Our results showed that plasma cortisol in both LT and HT group were dramatically elevated about 65–69% after 2 h thermal stress and declined with time to the control level at 8 d. Previous evidence showed that CRH played a key role in regulating the secretion of cortisol (Flik et al., 2006). However, UI and UII were also shown to stimulate the secretion of cortisol from isolated interrenal/head kidney preparations of seawater (SW)-adapted flounder (Kelsall and Balment, 1998), and UI also appeared to potentiate the steroidogenic actions of ACTH *in vitro* (Arnold-Reed and Balment, 1994). Therefore, plasma CRH and UII concentration were measured in the current study and it was found that the profile of plasma CRH matched closely with the trend in cortisol secretion but plasma UII did not respond to thermal stress. Studies pointed out that changes in CRH-related peptide secretion from the preoptic area (POA) and CNSS could impact upon the activity of the HPI axis (Huising et al., 2004; Bernier et al., 2008). As the CNSS is the major source of circulating CRH, UI and UII, we therefore, investigated the gene expression and secretion of these hormones from the CNSS.

Previous studies had found that thermal stress significantly changed CRH, UI, and UII expression at early developmental stage in zebrafish (Luo et al., 2014). In adult rainbow trout, salinity challenge produced a remarkable elevation in CRH and UI mRNA expression in CNSS (Craig et al., 2005). However, in this study, we found that CRH, UI, and UII mRNA levels exhibited different expression profiles not only as a result of the thermal conditions but also as a result of exposure time. Previous studies showed that additional UI and UII directly stimulate interrenal cortisol secretion in fish (Kelsall and Balment, 1998; Craig et al., 2005). The mRNA expression of CRH, UI and UII in CNSS was elevated after a 2 h period of hypothermal acclimation and returned back to the control level at 8 days. In contrast, gene expression of CRH, UI, and UII did not increase at the beginning under hyperthermal condition in CNSS. An increasing expression of CRH and UI was observed at 8 days hyperthermal treatment and a similar trend was seen in rainbow trout subject to a hyperammonemia condition (Bernier et al., 2008). The current mRNA expression results supported the view that CRH-, UI- and UII-expressing neurons in the CNSS could be recruited differentially and only in response to specific stimuli. From the expression profiles of these three genes, it is plausible that CRH, UI and UII in CNSS respond to acute hypothermal stress. By contrast, CRH and UI in CNSS did not respond to acute hyperthermal stress, but appeared to play an important role in hyperthermal adaptation, although the functional mechanism is less clear.

The number of Dahlgren cell were altered across seasons (Chen and Mu, 2008). However, until now, it was not known whether alterations in temperature would affect the number of Dahlgren cells and their associated secretions. Morphology and immunohistochemistry studies of the CNSS suggested that CRH, UI and UII were produced and secreted from the Dahlgren cells in the CNSS (Cioni and Bordieri, 2003; Lu et al., 2004). Therefore, the number of CRH-, UI- and UII-positive Dahlgren cells could represent the required secretory level of these genes in CNSS. In this study, we calculated the changes in UI:UII Dahlgren cell ratio and our results demonstrated that the percent of UI-positive Dahlgren cells increased after 8 d of hyperthermal treatment, and significantly higher than the NT group, with an additional match to the mRNA expression profile of UI in CNSS. No changes on percent of UI- or UII- positive Dahlgren cells were observed in the other treatment groups. To find out where the increasing percent of UI-positive Dahlgren cells came from, we used BrdU to label the new cells after thermal treatment and calculated the BrdU-labeled cells in CNSS. However, the number of BrdU-labeled cells in both LT and HT group were significantly lower than the NT group after 8 days thermal treatment. Additionally, no BrdU-labeled Dahlgren cells were observed in the CNSS (Figures 4B–D). Previous studies found that the neuroblasts lateral to the central canal were the precursor cells of Dahlgren cells (Fridberg, 1962). Therefore, the BrdU-labeled cells might partially be the regenerative neuroblasts, and the increased UI-positive Dahlgren cells might not be differentiated from newly regenerative neuroblast cells. Previous morphology and immunohistochemistry studies of the CNSS have found that CRH and UI were co-expressed (Cioni and Bordieri, 2003; Lu et al., 2004). Meanwhile, UI and UII could also be co-expressed in a fraction of Dahlgren cells (Parmentier et al., 2006). Therefore, the increased UI-positive cells might be recruited from the Dahlgren cells that expressed UII peptides. In brief, our results found that the percent of UI-positive Dahlgren cells increased after 8 days of hyperthermal treatment and the increasing percent of UI-positive Dahlgren cells might be due to the increased number of Dahlgren cells that co-expressed both UI and UII.

## Conclusion

In summary, this study demonstrates that the dynamic responses of hormone levels and the gene expression of CRH, UI and UII in the CNSS are associated with the whole process of hyper- or hypo-thermal stress response and adaptation. However, CRH-, UI- and UII-expressing neurons of the CNSS are recruited differentially and only in response to specific stimuli. Specifically, CRH, UI and UII in CNSS respond to acute hypothermal stress. By contrast, CRH and UI do not respond to acute hyperthermal stress, but appear to play a currently unknown role in hyperthermal adaptation. Our results indicate that the increase of UI-positive cells might originate from the increased Dahlgren cells that co-expressed both UI and UII during long-term hyperthermal adaptation in CNSS. Overall, our results supplements evidence that CNSS plays an important physiological role in thermal stress. Further research should focus on the specific thermal neural inputs that trigger the recruitment of CRH-, UI-, and UII-expressing cells in the CNSS.

## Data Availability Statement

All datasets generated for this study are included in the article/supplementary material.

## Ethics Statement

The experimental protocol was approved by the Institutional Animal Care and Use Committee (IACUC) of Shanghai Ocean University (SHOU), Shanghai, China, and abides by the Guidelines on Ethical Treatment of Experimental Animals established by the Ministry of Science and Technology, China.

## Author Contributions

WL and MY designed the study. MY and YC collated the data. MY, XL, and TL carried out the experiment, data analyses, and produced the initial draft of the manuscript. WL and MY contributed to drafting the manuscript. All authors participated in the manuscript revision, and have read and approved the final version of the manuscript.

## Conflict of Interest

The authors declare that the research was conducted in the absence of any commercial or financial relationships that could be construed as a potential conflict of interest.
